# Can CD133 Be Regarded as a Prognostic Biomarker in Oncology: Pros and Cons

**DOI:** 10.3390/ijms242417398

**Published:** 2023-12-12

**Authors:** Alisa Gisina, Yan Kim, Konstantin Yarygin, Alexey Lupatov

**Affiliations:** Laboratory of Cell Biology, V. N. Orekhovich Institute of Biomedical Chemistry, 119121 Moscow, Russia

**Keywords:** CD133, prominin-1, cancer biomarker, cancer prognosis, cancer stem cells

## Abstract

The CD133 cell membrane glycoprotein, also termed prominin-1, is expressed on some of the tumor cells of both solid and blood malignancies. The CD133-positive tumor cells were shown to exhibit higher proliferative activity, greater chemo- and radioresistance, and enhanced tumorigenicity compared to their CD133-negative counterparts. For this reason, CD133 is regarded as a potential prognostic biomarker in oncology. The CD133-positive cells are related to the cancer stem cell subpopulation in many types of cancer. Recent studies demonstrated the involvement of CD133 in the regulation of proliferation, autophagy, and apoptosis in cancer cells. There is also evidence of its participation in the epithelial–mesenchymal transition associated with tumor progression. For a number of malignant tumor types, high CD133 expression is associated with poor prognosis, and the prognostic significance of CD133 has been confirmed in a number of meta-analyses. However, some published papers suggest that CD133 has no prognostic significance or even demonstrate a certain correlation between high CD133 levels and a positive prognosis. This review summarizes and discusses the existing evidence for and against the prognostic significance of CD133 in cancer. We also consider possible reasons for conflicting findings from the studies of the clinical significance of CD133.

## 1. Introduction

**Figure 1:**
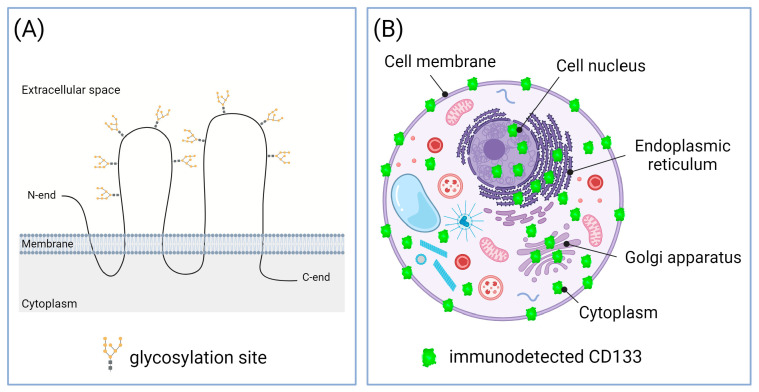
Structure and cellular localization of CD133. (**A**) Topological model of CD133 molecule at the plasma membrane. CD133 molecule has nine N-linked glycosylation sites [12]. (**B**) Subcellular localization of CD133. CD133 was detected in cell membrane; cytoplasm, including endoplasmic reticulum and Golgi apparatus; and nucleus. Created with BioRender.com.

The availability of reliable biomarkers for cancer screening and prognosis is essential for the early start of treatment and the achievement of more favorable outcomes. CD133, also termed prominin-1, is considered a promising prognostic biomarker for a wide range of tumor types. CD133 is an integral plasma membrane protein also detected inside the cell [1], including the endoplasmic reticulum and Golgi apparatus, where its maturation and glycosylation occurs [2]. The glycosylated CD133 has a molecular weight of ≈115–120 kDa [3,4], and in the plasma membrane, it is located predominantly at the membrane protrusions, such as microvilli and primary cilia [5]. Due to this, the name “prominin” comes from the Latin word “prominere”, which means “prominent”. The N-terminus and two large loops of this molecule are located outside the cell, and the C-terminus and two small loops are in the cytoplasm [6,7] (Figure 1A). It has been established that the CD133 glycoprotein on the plasma membrane resides in specialized lipid microdomains rich in cholesterol and sphingolipids, i.e., lipid rafts [8]. Recent studies have also revealed nuclear localization of CD133 [9,10,11]. Figure 1B shows all sites of the CD133 cellular localization.

CD133 is found in various tissues. In early embryos, CD133 is observed in trophoblast cells but not in the inner cell mass [13]. In developing embryos, CD133 is expressed in the epithelia of the three germinal layers [5,14]. In the postnatal period, CD133 expression occurs in stem and progenitor cells, such as hematopoietic stem cells [4], endothelial progenitor cells [15], prostate epithelial stem cells [16], brain stem/progenitor cells [17], myogenic cells [18], and hepatic progenitor cells [19]. CD133 is also expressed in differentiated cells of the adult body, and in particular, on various types of glandular epithelium and the epithelium of kidney proximal tubules [1,5,20,21]. CD133 is present on photoreceptor cells throughout human life, and mutations in the *PROM1* gene are associated with retinal degeneration leading to blindness [22,23].

In addition, CD133 is detected in many malignant tumors, such as prostate [24], lung [25,26], ovarian [27], breast [28], endometrial [29], renal [30], thyroid [31], esophageal [32], gastric [33,34], colorectal [35], pancreatic [1], hepatocellular [36,37], and gallbladder [38] carcinoma, as well as in glioma [39], meningioma [40], osteosarcoma [41], melanoma [42], cutaneous squamous cell carcinoma [43], and other solid malignant neoplasms. CD133 is also expressed on myeloid [44] and lymphoblastic leukemia cells [45]. Comparison of normal and tumor tissues revealed that the CD133 expression level in the vast majority of cases is significantly higher in the latter [25,46,47,48,49,50,51]. In addition, there is evidence that CD133 expression is higher in samples from patients with metastases and at the advanced stages of cancer [52]. There is an abundance of evidence of the existence of a relationship between the level of the CD133 protein or mRNA expression and the disease severity. However, there are also reports of the absence of such an association and even of a correlation with a favorable course of the disease. Thus, there is no final opinion with respect to the prognostic significance of CD133. For this reason, CD133 has not yet become a conventional prognostic cancer biomarker despite more than 20 years of intensive studies. The goal of the current review is to analyze the conflicting data regarding the prognostic value of CD133 and to highlight the possible reasons, including objective and methodological problems, leading to the observed contradictions.

## 2. Putative Explanation of the Cause-and-Effect Relationship between CD133 Expression and More Malignant Phenotype of Tumor Cells

The strong correlation between high CD133 levels in tumor cells and negative cancer prognosis complies with the association of CD133 expression with the cancer stem cell (CSC) phenotype. Initially, CSCs were identified as a subpopulation of tumor cells with an exceptional or increased ability to form tumors when transplanted into immunodeficient mice [53,54]. Later, it was shown that in addition to high tumorigenicity, CSCs are also characterized by increased invasiveness and metastasis, increased chemo- and radioresistance, higher proliferative activity, and the ability to form tumorspheres in vitro [55]. The role of CD133 as a CSC marker has been demonstrated in a wide range of human tumors. CD133 turned out to be effective for identifying CSCs in carcinomas of lung [25,26,56], ovary [57], stomach [58], colon [46,59], gallbladder [60], head and neck [61], pancreas [47], and liver [36], as well as glioblastoma [62], lymphoma [45], Ewing’s sarcoma [63], and in other human neoplasms [64,65]. Thus, high CD133 expression in biomaterial from a cancer patient may indicate the presence of a large number of CSCs with aggressive properties in the tumor.

### 2.1. Increased Proliferation

There is evidence of enhanced proliferation of tumor cells expressing CD133. For example, populations of CD133-positive cells from samples of oral squamous cell carcinoma [66] and cells of the hepatocellular carcinoma line [67] demonstrate a significantly higher proliferation rate than CD133-negative cells. This is consistent with our data obtained in colorectal adenocarcinoma cell lines, where the percentage of proliferating cells measured via BrdU incorporation, the number of visualized mitoses, expression of the proliferation marker Ki67, and the ability to form colonies in vitro were higher in populations with high CD133 expression compared with cells of the same lines with low or lacking CD133 expression [68,69]. There is also evidence that CD133-positive cells of osteosarcoma cell lines were mainly in the G2/M phase, expressed Ki67, and demonstrated higher proliferative activity compared to the CD133-negative cells, which were mainly in the G0/G1 phase and did not express Ki67 [70]. Also, there are data that the levels of surface CD133 fluctuate throughout the cell cycle in glioma cells with the highest level of CD133 found in S/G2/M [71]. In human and mouse neural stem cell lines, the CD133-negative cells were predominantly in G0/G1 phase, while CD133-positive cells mostly in S or G2/M phase [72].

**Figure 2:**
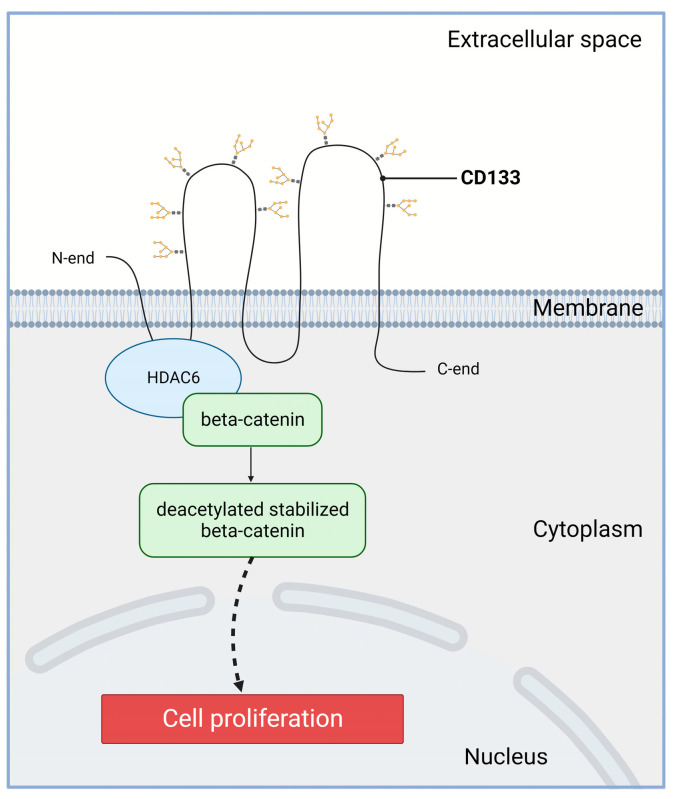
Involvement of CD133 in the Wnt/β-catenin signaling in cancer cells. Wnt—wingless-related integration site; HDAC6—histone deacetylase 6. The first intracellular loop domain of CD133 is able to physically bind to HDAC6 and β-catenin forming a ternary complex ensuring β-catenin stabilization. Created with BioRender.com.

However, it is important to note that the populations of proliferating cells and the populations of CD133-positive cells were never completely identical. Therefore, though CD133 is not a marker of proliferation, it is likely involved in this process. The molecular mechanism of the CD133-dependent stimulation of proliferation may be based on the activation of the Wnt/beta-catenin signaling pathway (Figure 2). Histone deacetylase 6 (HDAC6) is able to physically bind to both the first intracellular loop domain of CD133 and β-catenin stabilizing β-catenin in a ternary complex [73]. The CD133/HDAC6 complex induces deacetylation and inhibits proteasomal degradation of beta-catenin. Next, stabilized beta-catenin enters the nucleus and activates target genes activating cell proliferation and, accordingly, accelerating tumor growth. Reduced expression of CD133 or HDAC6 leads to increased degradation of beta-catenin, which correlates with a decrease in the proliferative activity of tumor cells [73]. Bisson et al. earlier reported on the clear association between Wnt signaling, beta-catenin nuclear localization, and CD133 expression in prostate cancer cells [74]. There is also another study indicating that activation of the Wnt/beta-catenin signaling pathway in CD133-positive liver cancer stem cells leads to their proliferation [75]. Furthermore, the hypoxia-inducible GLT8D1 (glycosyltransferase 8 domain containing 1) was shown to inhibit CD133 degradation in glioma CSCs [76]. Specifically, GLT8D1 interacts with the first extracellular domain of CD133 and prevents its lysosomal degradation through N-glycosylation and protein–protein interaction. As a result, the level of CD133 and β-catenin increases and stimulates WNT-β-catenin signaling [76]. Thus, CD133 may really enhance cancer cell growth through activation of the Wnt/beta-catenin signaling pathway.

Interesting data were obtained via the single-cell sequencing of colorectal cancer cells. It was shown that the transcriptome of all CD133-positive cells contained the stem-cell signature, but while some of the cells were in a dormant state, the remaining cells expressed the proliferating cells signature [77]. Quite probably, after the CD133-positive cell receives certain external signals, CD133 is involved in activating the proliferation process or, conversely, in bringing the cell into a state of quiescence. For example, it was recently found that under glucose deprivation, CD133 increases glucose uptake and stimulates autophagosome formation [78]. However, Izumi et al. found that when Src family kinase activity is low, CD133 interacts with HDAC6 and is transported to the pericentrosomal region after internalization and endosome formation via the dynein-based trafficking system. Pericentrosomal CD133 is then returned to the plasma membrane via recycling endosomes. In the pericentrosomal region, endosomal CD133 captures the autophagy initiator GABARAP and inhibits subsequent autophagy initiation. Moreover, pericentrosomal CD133 suppresses such hallmarks of cell differentiation as primary cilia formation and neurite outgrowth [79]. It seems that there is a relationship between the regulation of autophagy and asymmetric cell division mediated by CD133 endosomes [80]. Specifically, asymmetric distribution of the pericentrosomal CD133 endosomes in cooperation with nuclear beta-catenin causes unequal autophagy activity during cytokinesis in CD133-positive human neuroblastoma cells [81]. Thus, CD133 may be involved in the regulation of proliferation and dormancy depending on signals from the cell microenvironment. Although high proliferative potential rather than high proliferative activity is a hallmark of stem cells, the increased proliferative activity of CD133-positive tumor cells does not contradict their stemness. Whether stem cells are in a dormant state or actively proliferating depends on their niche and incoming external signals [82,83].

### 2.2. Increased Chemo- and Radioresistance

Multiple studies revealed correlation between the CD133 expression in primary carcinomas and the resistance to chemotherapeutic drugs [84]. It was shown that CD133-positive cells of oral squamous cell carcinoma were more resistant to cisplatin [66]. Silencing of CD133 in colorectal cancer cells lead to their high sensitivity to oxaliplatin [85]. CD133-positive glioblastoma cells have been reported to be more resistant to various drugs, including temozolomide, carboplatin, paclitaxel, and etoposide [86]. In our in vitro experiments, we found a correlation between the chemoresistance of colorectal cancer cells HT-29 and the expression of CD133. The HT-29 subline, enriched by FACS by the cells with high CD133 expression level, showed higher resistance to the protein kinase inhibitors sorafenib, sunitinib, temsirolimus, and everolimus, compared with the subline that did not contain CD133-expressing cells. A change in the sensitivity of tumor cells to mTOR inhibitors, temsirolimus and everolimus, was also found in HT-29 cells with a complete knockout of the *PROM1* gene [87]. It was also shown that in tumor cells isolated from primary glioblastomas, there was an enrichment of the CD133-positive cells after exposure to ionizing radiation in vitro and in vivo, which was combined with enhanced DNA repair and reduced sensitivity to radiation-induced apoptosis [39]. Another study provided evidence that CD133 may influence the efflux of chemotherapy drugs from cancer cells [88]. Specifically, the chemoresistant KOA-1 subline was found to have significantly increased CD133 mRNA expression, increased cell survival and invasion capacity, and increased expression of the drug resistance proteins MDR1 and MRP1 and corresponding mRNAs. Moreover, inhibition of CD133 expression by siRNA led to a decrease in the expression of mRNA and drug resistance proteins MDR1 and MRP1 [88]. There is also evidence that the population of CD133-positive melanoma cells is specifically enriched in cells expressing the ABCB5 transporter, which is responsible for pumping physiological metabolites and xenobiotics out of the cell [89].

**Figure 3:**
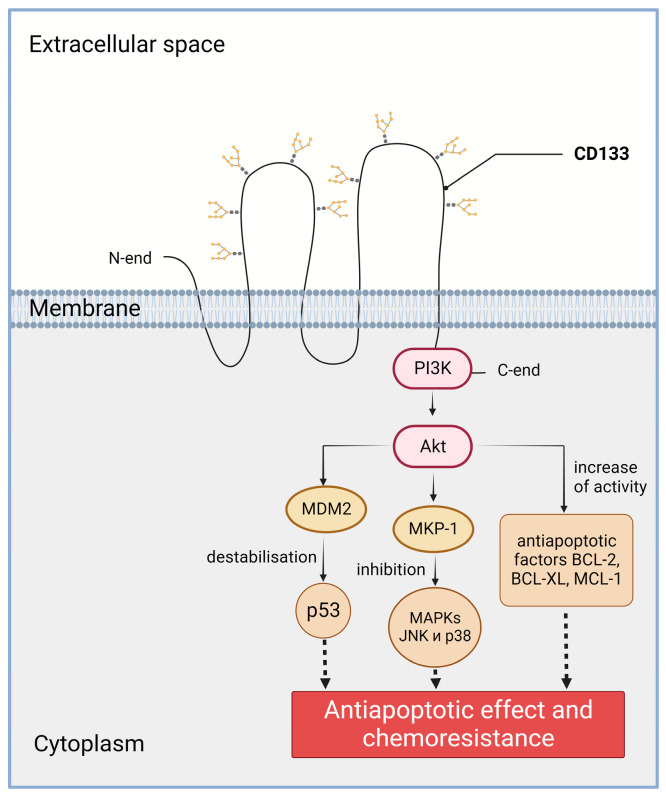
Involvement of CD133 in the PI3K/Akt signaling pathway in cancer cells. PI3K—phosphoinositide 3-kinase; Akt—RAC-alpha serine/threonine-protein kinase (a.k.a. protein kinase B alpha); MDM2—E3 ubiquitin-protein ligase Mdm2; p53—transformation-related protein 53; MKP-1—protein kinase phosphatase-1; MAPKs—mitogen-activated protein kinases; JNK—c-Jun-NH(2)-terminal kinase; BCL-2—B-cell lymphoma 2 protein, apoptosis regulator; BCL-XL—anti-apoptotic protein, BCL2 family member; MCL-1—apoptosis regulator, BCL2 family member. The C-terminal cytoplasmic domain of CD133 is able to physically bind to PI3K. Created with BioRender.com.

The role of CD133 in the chemo- and radioresistance of tumor cells may be related to the ability of CD133 to activate the PI3K/Akt signaling pathway (Figure 3). CD133 has a short C-terminal cytoplasmic domain with five tyrosine residues, including a tyrosine phosphorylation site. When studying glioma cells, it was found that phosphorylation of the tyrosine-828 residue of CD133 leads to activation of the PI3K (phosphoinositide 3-kinase) pathway through direct interaction of CD133 and the 85kDa subunit of PI3K [90]. Specifically, in CD133-positive cancer cells, Src kinase phosphorylates tyrosine-828 in the C-terminal cytoplasmic domain of CD133. The phosphotyrosine-828 residue interacts with p85, leading to the activation of the p110 catalytic subunit of PI3K. Activated PI3K converts PIP2 (phosphatidylinositol 4,5-bisphosphate) to PIP3 (phosphatidylinositol 3,4,5-trisphosphate). PIP3 then induces the phosphorylation and activation of Akt (also known as protein kinase B) [90]. There is also evidence that CD133 knockdown inhibits PI3K/Akt activity and increases the survival of mice in tumor cell xenotransplantation tests. CD133 activates the PI3K signaling pathway and, as a result, Akt is also activated. Activation of Akt, in turn, leads to increased activity of the BCL-2, BCL-XL, and MCL-1 anti-apoptotic factors [90]. In addition, knockdown and overexpression experiments showed that CD133 prevents the death of colon cancer cells from nutrient deprivation through the activation of the Akt-mediated anti-apoptotic signaling pathway [91]. Functional analysis of the CD133-positive and CD133-negative cells in vitro and in vivo revealed that melanoma progression and therapy resistance are a consequence of CD133 signaling in the PI3K pathway. The CD133 signal in the PI3K pathway triggers two downstream signaling cascades—PI3K/Akt/MDM2 and PI3K/Akt/MKP-1. Activation of the PI3K/Akt/MDM2 signaling pathway leads to the destabilization of the p53 protein, while activation of the PI3K/Akt/MKP-1 signaling pathway leads to the inhibition of JNK and p38 MAPKs. Activation of both signaling pathways leads to the inhibition of fotemustine-induced apoptosis. Thus, the disruption of CD133 signaling in the PI3K signaling pathway is required to overcome melanoma resistance to fotemustine [92].

### 2.3. Increased Invasion and Metastasis

Epithelial–mesenchymal transition (EMT) is the process of acquiring the mesenchymal cell phenotype by the epithelial cells. It can occur under normal physiological conditions, for example, during embryogenesis, organ formation, and wound healing [93], or during carcinogenesis [94]. Since after EMT tumor cells become more malignant, i.e., prone to tissue invasion and metastasizing [95], the role of EMT in carcinogenesis has been extensively studied, including the assessment of the involved molecules, such as E-cadherin, N-cadherin, vimentin, and others [94].

In the experiments with HT-29 cells, it has been shown that the knockdown of the epithelial marker E-cadherin, downregulated during EMT, enhances cell migration and invasiveness, upregulates EMT-associated proteins, promotes morphological transition towards mesenchymal phenotype, and boosts the expression of CSC markers CD44 and CD133 [96]. There is also evidence that CD133 itself can influence the EMT process. Thus, its knockout in LoVo colorectal cancer cells leads to a decrease in cell migration ability and invasiveness, as well as loss of the expression of vimentin, usually upregulated during EMT [97]. Moreover, in the highly migratory pancreatic cancer subline, Capan-1 CD133 appears to be critical for the EMT efficacy because its binding to ERK1/2 and SRC is the key step for initiating N-cadherin expression [98]. In the same subline, the knockdown of CD133 leads to a decrease in the expression of the transcription factor Slug (SNAI2) responsible for EMT, as well as a decrease in the migratory capacity and invasiveness of cells [99]. Compared to the maternal cell line, this subline shows increased CD133 expression, as well as certain mesenchymal cell features, such as increased expression of the transcription factors Slug (SNAI2) and Snail (SNAI1), N-cadherin, and fibronectin, as well as decreased expression of occludin and desmoplakin. Similar data were obtained using the cells of an oral cancer line, in which ectopic CD133 overexpression enhanced the expression of Oct4, Sox2, and Nanog stem cell markers, as well as N-cadherin and vimentin, and, at the same time, stimulated cell migration and invasiveness [100]. CD133 appears to be involved in the EMT regulation also through paracrine effects. Specifically, CD133-positive ovarian cancer cells, through the secretion of CCL5, induce enhanced metastatic capacity in the CD133-negative cells in vitro and in vivo [101]. The important function of CD133 in the regulation of EMT was also demonstrated in the non-small cell lung cancer line A549. Although TGF-b1 induced EMT in all cells of this line, only CD133-positive cells, when exposed to this factor, increased their migratory potential and the expression of another EMT marker—MMP9 [102]. Obviously, CD133 is an important regulator of the EMT.

The correlation between the CD133 level, EMT markers, and the clinicopathological characteristics, such as invasiveness and metastases, was also established in surgical biopsies taken from patients. In isolated breast cancer cells, CD133 expression directly correlated with that of N-cadherin typically expressed in the mesenchymal cells [103]. Notably, in metastases, the expression of N-cadherin was higher compared to the primary lesions. In line with this, CD133 expression was found to inversely correlate with the epithelial E-cadherin in the specimens of ovarian cancer [103], intrahepatic cholangiocarcinoma [104], colorectal cancer [105], and lung cancer [106]. Also, some of these papers demonstrated direct correlation between CD133, EMT markers N-cadherin and vimentin, the level of metastases, and poor prognosis. The correlation between CD133 and EMT-related markers is also observed in physiological conditions. For example, CD133 expression correlates with E-cadherin loss from hair follicle placodes during morphogenesis [107].

Using the samples from patients with colorectal adenocarcinoma, we found a strong correlation of CD133 expression with the overexpression of another cell adhesion molecule, CEACAM5, considered the best indicator of local cancer recurrence and distant metastases [108]. Indeed, recently, it was shown that CEACAM5 is involved in regulation of EMT [109].

Thus, there is an abundance of evidence demonstrating the involvement of CD133 expression in the EMT process. Apparently, CD133 is its direct participant, and CSCs expressing this protein acquire an aggressive phenotype due to increased mobility, invasiveness, and metastasis.

### 2.4. CD133 as a Target of Therapies Aimed to Eliminate the Most Malignant Cells

The role of CD133 in tumor progression is further evidenced by the results of the preclinical and clinical studies of antitumor drugs targeting the CD133 molecule (for more information, see reviews [110,111]). Such approaches include delivery systems for conventional chemotherapy drugs, as well as drugs that activate the cytotoxic mechanisms of the immune system. In particular, a chimeric drug that is a CD133-targeted DNA aptamer combined with doxorubicin showed selective killing effect in human colorectal cancer HCT116 cells expressing CD133. The in vitro and in vivo results demonstrated the high therapeutic efficacy and low toxicity of this chimera [112]. Also, recent in vitro study has shown the effective use of Fc-optimized antibodies against CD133 to induce natural killer (NK) cell reactivity [113]. In this work, engineered mAbs with Fc parts that display enhanced affinity to the CD16 Fc receptor expressed on NK cells were used as drugs in order to improve antibody-dependent cellular cytotoxicity against CD133-expressing cells. The authors demonstrated that the developed Abs specifically induced activation of NK cells resulting in the lysis of the B-cell acute lymphoblastic leukemia cells [113]. The greatest progress has been made in the development of the targeted anti-CD133 therapy based on chimeric antigen receptor-modified T-cell (CAR-T) cells. For example, Wang et al. reported the use of autologous CD133 targeted CAR-T cells in Phase-I trial, completed in 23 patients with late-stage metastasis malignancies (14 with hepatocellular carcinoma, 7 with pancreatic carcinomas, and 2 with colorectal carcinomas). The anti-tumor specificity and toxicity of CD133-targeted CAR-T cells were assessed. Using biopsy material, CAR-T cells were shown to destroy CD133-positive cells. Of 23 patients, 3 achieved partial remission and 14 achieved disease stabilization for 9 weeks to 15.7 months. No de novo lesions were detected in 21 out of 23 patients following administration of CAR-T cells during the follow-up period [114]. Approaches combining the use of CD133-targeted CAR-T cells with chemotherapy are also being developed. For example, a combination therapy of cisplatin and anti-CD133 CAR-T cells aiming to selectively target cisplatin-resistant CSCs showed effectiveness in a gastric cancer model [115]. Specifically, the cisplatin and anti-CD133 CAR-T combination treatment inhibited tumor progression in three different xenograft models and diminished CD133-positive cell infiltration [115]. Another way to enhance the efficacy of CAR-T therapy is to use additional target molecules. For example, AC133-specific CAR-T cells used alone reduced the tumor burden and prolonged survival in a humanized orthotopic small cell lung cancer model, but were not able to entirely eliminate tumors, whereas the triple immunotherapy combining PD-1 and CD73 inhibition with CAR-T cell treatment cured 25% of the mice without signs of graft-versus-host disease or bone marrow failure [116]. Singh and colleagues generated a dual-antigen T cell engager based on an antibody fragment that targets a unique epitope present in glycosylated and non-glycosylated CD133, as well as CD133-specific CAR-T cells. All three modalities were efficacious in orthotopic glioblastoma xenografts against patient-derived CD133-positive glioblastoma cells. However, predictably, CAR-T cells demonstrated superior efficacy [117].

## 3. Association of CD133 with Cancer Progression and Poor Prognosis

The direct correlation between CD133 expression on one hand and various clinicopathological parameters, such as overall survival, tumor stage and differentiation level, metastasis, and recurrence rate on the other, has been demonstrated for many types of malignancies. Moreover, a number of the meta-analyses have shown a correlation between CD133 expression levels and a more severe course of cancer (Table 1).

Despite the convincing evidence of the association between CD133 expression and the unfavorable cancer progression, the opposite was also reported. There are data indicating the absence of such a correlation or even an association of CD133 expression with a favorable disease prognosis. For illustration, in the immunohistochemical analysis of 88 glioblastoma samples, the presence of CD133 was detected in 52 cases, but a comparative analysis of the studied glioblastoma cases did not reveal a statistically significant association between the presence of CD133 and patient survival [143]. Interestingly, in this study, CD133 was rarely detected at the membrane, but mostly showed dense cytoplasmic staining with small granular patterns in tumor cells. At the same time, CD133 expression was also detected in the endothelium. Another study using 78 glioblastoma samples from patients treated with temozolomide and radiotherapy, also failed to prove CD133’s value as a prognostic marker of patient survival [144]. A retrospective analysis of 114 deparaffinized astrocytoma specimens found no correlation of CD133 expression with tumor stage or patient survival [145].

A recently published study analyzed CD133 mRNA expression in 60 colon cancer samples and found no correlation of its expression with the aggressive phenotype of primary and metastatic tumors. In primary tumors, CD133 mRNA expression did not correlate with aggressive phenotypes, and in liver metastases, it was significantly lower compared with primary tumors [146]. In another colorectal cancer study, the immunohistochemical analysis of the CD133 expression in 142 primary and 75 peritoneal lesions identified CD133 in 55% and 40% of the tumor samples, respectively. CD133 expression was not found to be associated with overall patient survival. Moreover, the disease-free survival of patients was higher in the CD133-positive group compared with the CD133-negative group. Interestingly, in this study the patient benefit from systemic chemotherapy was significantly greater in the CD133-negative group [48]. Since, as noted above, CD133 may contribute to cell chemoresistance, this result might be expected. However, in another study, Mia-Jan et al. obtained opposite results [147]. They examined 271 samples of stage II and III colorectal cancer, including 171 samples from patients who underwent adjuvant chemotherapy after surgery and 100 samples from patients without adjuvant therapy. Surprisingly, they found that among patients who received adjuvant therapy, CD133-positive tumors were associated with longer overall survival [147]. In a recently published study Yamanaka et al. conducted an immunohistochemical analysis of CD133 expression in breast cancer samples from 55 patients and found no differences in the level of expression of this marker between primary and metastatic tumors [148]. The authors found that patients with high CD133 expression had lower disease-free and overall survival. At the same time, the authors did not find an association between CD133 expression in metastatic tissues and survival.

The conflicting results may be caused by such factors as different patient cohort sizes, varying cutoff values of positive CD133 expression or alternative approaches to the treatment. For example, two studies analyzing the correlation of the CD133-positive status of endometrial carcinoma with prognosis reported quite opposite results [149,150]. This particular controversy may be linked to different assessments of the level of the CD133 expression (in one study, samples were considered CD133-positive if more than 1% of cells expressed CD133, and in the second, if more than 10% of cells expressed CD133). Also in these studies, the patient cohort demonstrated different mean rates of disease-free survival and overall survival [149]. Meta-analysis of the combined data taken from many studies sometimes allows us to level out such differences between studies due to large sample size. However, for some tumors, including renal cell carcinoma and esophageal cancer, a correlation between CD133 expression and the unfavorable disease course was not found even in meta-analyses [127,131] (Table 1).

## 4. Possible Reasons for the Discrepancies in the Data on the Association of CD133 with the Disease Severity

### 4.1. Different CD133 Immunodetection Techniques

The results of the CD133 detection in different studies may vary due to the variations in the methods of tumor tissue preparation for immunohistochemical staining. CD133 is known to bind to cholesterol [151] and possibly to gangliosides within lipid rafts on a plasma membrane [152,153,154]. Most raft proteins, such as glycosylphosphatidylinositol (GPI)-anchored proteins, are insoluble in Triton X-100 detergent used for membrane permeabilization due to their tight packing with cholesterol and glycolipids [155,156]. However, it was found that CD133-containing rafts could be dissolved in Triton X-100 [157]. Thus, the use of this detergent, for example, in intracellular staining, can wash CD133 away from the cell membrane, reducing its level. An alternative may be Lubrol WX, which does not dissolve the CD133-containing rafts [157].

It is known that cross-links in proteins formed as a result of exposure to formaldehyde can lead to changes in their topology and difficulty in recognizing certain epitopes by antibodies [158]. In particular, this previously impeded accurate detection of the proliferation marker Ki-67, commonly used in the clinic, in paraffin sections [159,160]. There is also an assumption that xylene, used in the deparaffinization of archival tumor tissue samples, can alter the spatial structure the membrane proteins concealing certain epitopes [161]. In some cases, application of the antigen retrieval methods improving the accessibility of epitopes after the fixation and deparaffinization procedure significantly increase the immunoreactivity of proteins [158]. Unexpected findings were reported by Kemper et al., who showed that compared to gradual freezing of the tissue in nitrogen vapor followed by treatment with acetone, the CD133-specific immunoreactivity of the tissue was higher if frozen tissue was treated with paraformaldehyde [162]. It is possible that in some cases, fixation may not mask the epitope but improve its accessibility to the antibodies. In this regard, the use of the antigen retrieval techniques should be assessed when using new antibody clones [163].

**Figure 4:**
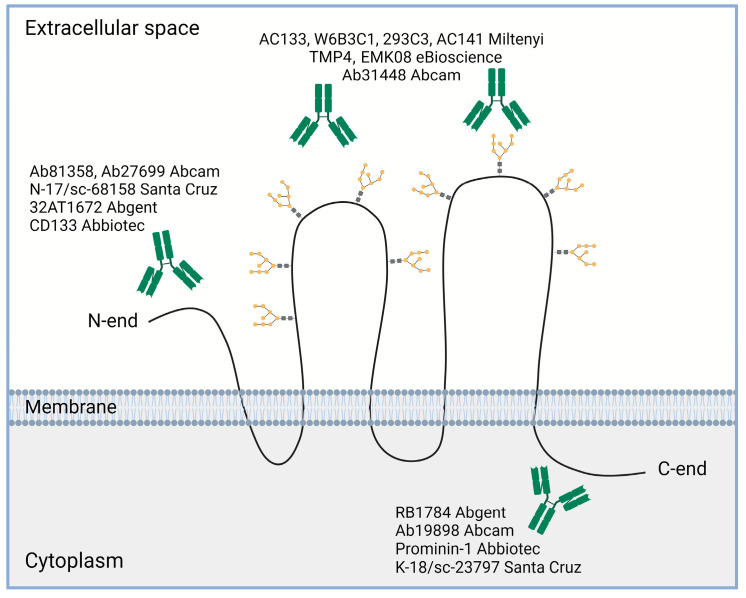
Some examples of commercial antibodies used to recognize different domains of CD133 molecule (based on review [84]). Created with BioRender.com.

Importantly, since the initial identification of the CD133 molecule using the mouse monoclonal antibody AC133 [4], a large panel of commercial and custom antibodies was created that recognize various parts of the CD133 molecule [84] (Figure 4). Among them, the AC133, W6B3C1, 293C3, AC141 (Miltenyi, Bergisch-Gladbach, Germany), TMP4 and EMK08 (eBioscience, San Diego, CA, USA), and Ab31448 (Abcam, Cambridge, UK) antibodies are suitable for detecting the extracellular loops of the CD133 molecule; the CD133 (Abbiotec, Escondido, CA, USA), Ab81358 and Ab27699 (Abcam), 32AT1672 (Abgent, San Diego, CA, USA), and N-17/sc-68158 (Santa Cruz, Santa Cruz, CA, USA) antibodies are used to reveal the N-terminal domain of the CD133 molecule; and the Prominin-1 (Abbiotec), RB1784 (Abgent), K-18/sc-23797 (Santa Cruz), and Ab19898 (Abcam) antibodies bind to the C-terminal domain. A more complete list of existing anti-CD133 antibodies is presented in the review by Grosse-Gehling et al. [84]. Moreover, articles describing the development of new antibodies to CD133 are also periodically published [164,165,166,167,168,169]. In the case of antibodies to epitopes on extracellular loops, the question often arises as to which epitope they detect—glycosylated or non-glycosylated. Such studies have been carried out for a number of antibody clones. For example, in a study using recombinant CD133 expressed in bacteria, it was shown that antibody clones AC133, W6B3C1, AC141, 293C recognized non-glycosylated epitopes on the second extracellular loop of the recombinant CD133 protein [162]. This does not mean that the same antibodies are not good for recognizing glycosylated CD133 where the corresponding epitopes are available. However, it should be taken into account that glycans in the CD133 molecule make a significant contribution to the specific configuration of epitopes recognized by different antibody clones resulting in a heavy impact of the CD133 glycosylation upon the binding of some antibodies [170]. Inaccurate CD133 assessment may occur in non-homeostatic conditions, since a noticeable change in CD133 glycosylation may occur as a result of hypoxia [76]. Really, several studies have shown that CD133 expression increases in glioma cells when cultured under hypoxic conditions [171,172,173,174,175], but Lehnus et al. obtained the opposite result [176]. This contradiction can probably be explained by the fact that antibodies of different clones were used in these studies. Increased expression of CD133 in response to hypoxia was detected using antibody clones AC133 [171,172,174,175,177] and 293С3 [171], while decreased CD133 expression was detected using the W6B3C1 clone [176].

To make things more complicated, the *PROM1* has five different promoters that are regulated in a tissue-dependent manner, leading to the expression of alternative CD133 splice variants [178], including a splice variant with truncated C-terminal domain [179]. Alternative splice variants may have a varying topology within the plasma membrane, also impairing their recognition by some antibodies. Apparently, the truncated splice variant will not be recognized by antibodies targeting the C-terminal domain of CD133.

Another complication comes from the cross-reactivity of AC141 antibodies with cytokeratin 18 presented on the epithelium [180], as well as the cross-reactivity of AC133, C24B9, and ab19898 antibodies with human serum within blood vessels [163]. Additionally, when working with cell cultures, factors such as the amount of serum [181], mycoplasma contamination [182], and the degree of confluence [14] may affect the CD133 expression profile. In summary, we can conclude that careful antibody testing and using a combination of antibodies to different parts of the CD133 molecule can help more accurately assess the expression of this molecule.

### 4.2. Some CD133-Positive Cells Present within Tumors Are Normal, Benign Cells

**Figure 5:**
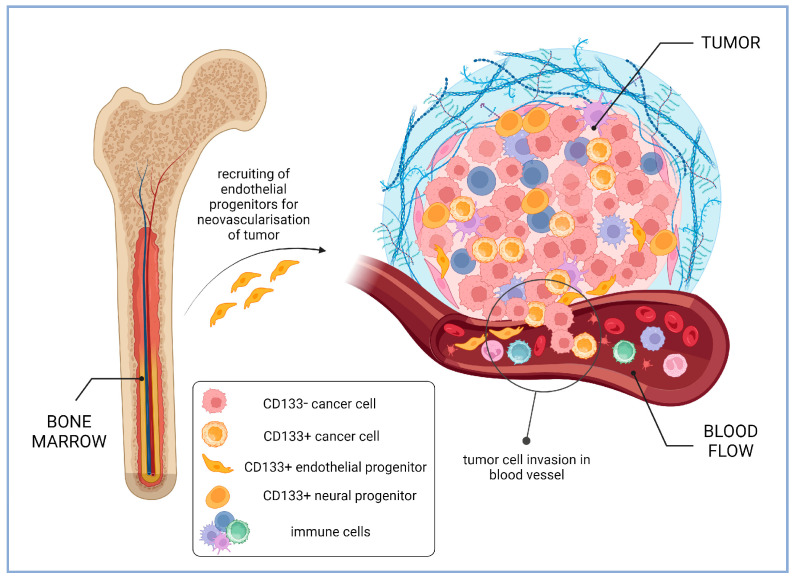
Intra-tumoral and circulating CD133-positive cells in cancer patients (based on the results of glioma studies). Higher percentage of CD133-positive endothelial progenitors recruited from bone marrow for tumor neovascularization is associated with lower patient survival [189]. Perivascular niches enriched with CD133-positive cells are several times more numerous in the higher stage gliomas [145]. CD133-positive neural progenitors in glioblastomas may contribute to a more favorable course of cancer disease [187]. Created with BioRender.com.

Since endothelial progenitors and other stem and progenitor cells express CD133, their integration in tumor tissue can be wrongly interpreted as the presence of CSCs. For instance, in study of glioma samples, it was revealed that CD133-positive cells present in low-grade gliomas were predominantly endothelial cells expressing CD31, while in glioblastoma samples, the majority of CD133-positive cells were of tumor origin, although CD31+CD133+ cells were also detected. It was found that the CD133 level on the surface of CD45-CD31- tumor cells inversely correlated with the patient’s survival time [183]. Perivascular niches are the predominant site of the CD133-positive cells’ localization within gliomas, and those of them enriched with CD133-positive cells are several times more numerous in the higher stage tumors [145] (Figure 5). However, analysis of the correlation between CD133 expression in gliomas with patient survival rate did not reveal prognostic significance of either the total number of CD133-positive cells within the niches or the number of the CD133-positive cells in the vasculature. In this work the result may have been influenced by the sample processing method (samples were embedded in paraffin and not frozen) and also by the use of the W6B3C1 monoclonal antibodies to detect CD133 [145]. On one hand, the used approach made it possible to detect perivascular niches, since formalin fixation and paraffin preserved the tissue structure; but on the other hand, the proportion of CD133-positive tumor samples was lower than in other similar studies probably due to inappropriate methodology.

The benign, non-tumor CD133-positive cells can contribute to tumor progression. It was reported that, in vitro, the CD133-positive hematopoietic progenitor cells from the umbilical cord blood can invoke additional malignancy in breast cancer cells, blocking spontaneous apoptosis and stimulating the process of the epithelial–mesenchymal transition [184]. The tumor-activating effect of the CD133-positive endothelial progenitors may also explain communications stating that the VEGF-stimulated CD133-positive cancer cells induce the relapse of the hepatocellular carcinoma [185] or the migration/invasion of salivary adenoid cystic carcinoma cells [165] by inducing the vasculogenic mimicry formation. It has also been shown that CD133-positive cells have a higher ability to migrate and invade and also demonstrate higher levels of endothelial cell markers VE-cadherin, MMP-2, and MMP-9 [186].

Interestingly, CD133-positive cells of non-tumor origin can also inhibit tumor growth [187]. In a study of the paraffin-embedded glioblastoma samples, it was shown that during relapses the percentage of the CD133-positive cells can greatly increase sometimes 10- or even 20-fold. Surprisingly, higher CD133 expression at relapse correlated with longer survival. The in-depth analysis showed that the population of the CD133-positive cells included not only cancer cells but also 20–60% of normal neural progenitor cells. Apparently, it is this population that contributes to a more favorable course of recurrent disease [187] (Figure 5). However, the percentage of the detectable CD133-positive cells in cultures isolated from glioblastoma samples and the percentage of the CD133-positive cells co-expressing the proliferation marker Ki67 inversely correlate with overall and relapse-free survival of patients [188]. Thus, not all but only actively dividing CD133-positive cancer cells contributed to the unfavorable course of the cancer disease.

Not only histological samples of the tumor but also blood samples from patients are used to establish correlations between the level of CD133 and the severity of the disease. Thus, it was found that CD133 expression level in the blood of patients with liver metastases from colorectal cancer correlates with survival [190]. This may be due to the circulation of CSCs in the blood of patients. It was also found that the level of CD133 mRNA detected in peripheral blood mononuclear cells from patients with colorectal cancer was higher in recurrent disease patients [191]. An important feature of such studies involving the quantitative determination of mRNA is the inability to track its cellular source. The number of CD133-positive cells in blood may be associated not only with the circulation of cancer cells in the blood but also with the recruitment of endothelial progenitor cells from the bone marrow for tumor neovascularization [192]. For example, it was reported that the CD133+/VEGFR-2+ circulating endothelial progenitor cells are more abundant in patients with glioblastomas compared to low-grade gliomas, and their higher percentage is associated with lower patient survival [189] (Figure 5). In practical terms, however, these data are very useful in any case because they indicate that CD133 mRNA may be a potential prognostic marker even if its origin is obscure.

### 4.3. Subcellular Localization of CD133 May Vary at Different Stages of Carcinogenesis

It has long been known that CD133 is not only associated with the cell membrane; it can also be found in the cytoplasm, including the endoplasmic reticulum and the Golgi apparatus, where its maturation occurs [2,193]. Nuclear localization of CD133 has also been demonstrated, for example, in the immunohistochemical studies of hepatocellular carcinoma samples [194] and samples of triple-negative breast cancer [10]. Nunukova et al. found atypical nuclear localization of CD133 in five rhabdomyosarcoma cell lines [9]. Moreover, a small subpopulation of cells exhibiting exclusively nuclear localization was identified. The authors verified the nuclear localization of CD133 using different types of antibodies to the extracellular loops and the C- and N-termini, and confocal as well as transmission electron microscopy. Additionally, they performed cellular fractionation followed by immunoblotting. All obtained data confirmed the presence of CD133 in the nucleus. Also, the authors noted that in addition to cells with typical membranous and exclusively nuclear localization of CD133, there were also sporadic clusters of positive signaling in the cytoplasm near the cell nucleus or very close to the nuclear envelope [9]. In a study of samples of tumor tissue and corresponding adjacent normal tissue from 239 patients with non-small cell lung cancer, nuclear localization of CD133 was also detected in addition to the cytoplasmic localization [49]. Both cytoplasmic and nuclear CD133 protein expression levels were significantly higher in tumor tissue compared to the corresponding peritumoral tissue. The expression of CD133 in the nuclei of tumor cells correlated with tumor diameter, degree of tumor differentiation, and the stage of the disease. Moreover, it was found that high nuclear CD133 expression and high cytoplasmic CD133 expression were prognostic factors for poor outcome of non-small cell lung cancer [49]. Pietrus et al. investigated the association of subcellular expression of CD133 with the clinical manifestations and outcomes of the endometrial cancer [11]. They performed an immunohistochemical study of CD133 expression in the plasma membrane, nucleus, and cytoplasm in a group of 64 patients with endometrial cancer and found that CD133 nuclear expression is directly associated with the stage of disease. Specifically, CD133 nuclear expression was increased in stages IB-IV compared to stage IA [11]. In hepatocellular carcinoma patients, nuclear CD133 expression was an indicator of poor prognosis: median survival span 12 months versus 34.5 months for patients with no nuclear CD133 expression [195]. However, Chen et al., studying the influence of subcellular localization of CD133 on the prognosis of patients with hepatocellular carcinoma, obtained a different result [196]. The authors performed immunohistochemical analysis of CD133 expression in 119 tumor tissue samples and matched normal liver tissue samples from patients with hepatocellular carcinoma who had not received chemotherapy or targeted therapy before surgery. It was found that CD133 expression levels in both the cytoplasm and nucleus were significantly higher in tumor tissue than in adjacent normal liver tissue. Analysis of the correlation of CD133 expression level with disease prognosis showed that high CD133 expression in the cytoplasm was an independent factor of poor prognosis. However, unexpectedly, high expression of CD133 in the nucleus of hepatocellular carcinoma cells turned out to be an independent factor for a favorable prognosis of overall and relapse-free survival [196]. Similar data were obtained in the study on colorectal adenocarcinoma. Specifically, nuclear localization of CD133 was shown to correlate with good prognosis [197]. In addition, both cytoplasmic and nuclear expression of CD133 were found to be significantly lower in adenomas than in colorectal adenocarcinoma. Interestingly, cells of the tumor microenvironment in adenomas showed higher expression of CD133, which may indicate their special role in the development of the tumor process. It was also found that in samples of colorectal cancer metastases, the levels of the cytoplasmic and nuclear expression of CD133 were reduced in both cancer cells and cells of the tumor microenvironment compared with samples from primary lesions. At the same time, the nuclear expression of CD133 in the cells of the primary lesion of colorectal cancer and the expression of CD133 in the cells of the tumor microenvironment were reduced in patients with a poor prognosis. These observations suggest a decrease in CD133 expression at the late stages of carcinogenesis, which may be associated with the loss of prognostic significance of cytoplasmic and nuclear expression of CD133 at the late stage of carcinogenesis [197]. Thus, the subcellular localization of CD133 presumably indicates a certain stage of carcinogenesis, and its prognostic significance remains to be investigated.

## 5. Conclusions

When CD133 was first suggested as a CSC marker, little was known about its function and properties. However, recent studies proved the involvement of CD133 in the regulation of proliferation, autophagy, apoptosis, and epithelial–mesenchymal transition in cancer cells. This new evidence suggests that CD133 can be a good prognostic tool for some cancer patients, since it is involved in defining the CSC phenotype of cancer cells. The comprehensive review of meta-analyses presented here confirms the correlation between CD133 expression and certain signs of the disease severity in many malignancies including the following: osteosarcoma, glioma, colorectal cancer, head and neck squamous cell carcinoma, hepatocellular carcinoma, non-small cell lung cancer, ovarian cancer, pancreatic ductal adenocarcinoma, gastric cancer, and breast cancer.

Despite the abundance of data concerning the prognostic significance of CD133, this molecule has not yet found its place in clinical oncology as a biomarker. Probably, translation of the research results into clinical practice is complicated by the following reasons. First, not all CSCs express CD133. Some tumor cells maintain CSC phenotype utilizing alternative regulatory mechanisms, not involving CD133. Second, CD133 is expressed by some normal, not malignant cells present within the tumor lesion. Third, CD133 expression by CSC can be transient and depends upon the cellular microenvironment (niche) [198] or other internal and external factors like hypoxia [199]. Fourth, CD133 expression and its impact on the tumor progression may differ at the different stages of tumor growth. In addition, methodological factors such as different antibody clones and tissue preparation techniques may cause differences in test results. All these issues may lead to inconsistencies in the original studies and cause the display of conflicting results and conclusions in reviews and meta-analyses.

In order to accelerate adoption of CD133 as a biomarker, tumor type (subtype), stage, and the evaluation method must be clearly defined. This will require further large-scale studies taking into account new data on genetic differences between tumor subtypes and a standardized technical approach to biomarker detection. The stage of the disease is important for accurate assessment of the outcomes because as tumor progression occurs, the influence of factors other than tumor cell phenotype on survival is expected to increase dramatically. For this reason, starting from the initial stages long-term follow-up will be required. Since populations of benign cells expressing CD133 can contribute to cancer progression, the study must separately consider quantitative information about these cell types. It also seems promising to take into account the prognostic significance of the intracellular localization of CD133. Such features of the study as inappropriate types of antibodies used or too-harsh processing of tumor tissue before immunohistochemical analysis can significantly misrepresent the picture of the actual expression of CD133 in the cell. A possible solution to this problem would be to use a set of antibodies for different parts of the CD133 molecule to select the clone with the highest prognostic value. All this can be taken into account, and CD133 will eventually be introduced as one of the prognostic biomarkers. Moreover, it can be regarded as a potential target for antitumor drug development.

## Figures and Tables

**Table 1:** An overview of the meta-analyses analyzing the clinicopathological and prognostic significance of CD133 in cancers. PubMed query: “CD133[title/abstract] OR prominin-1[title/abstract] OR PROM1[title/abstract]” with an article type set on “Meta-analysis” (https://pubmed.ncbi.nlm.nih.gov, entry date 20 November 2023).

Tumor Type	Reference	Analyzed Studies	Total Cancer Cases	CD133 Detection Method	Correlations *
Osteosarcoma	[118]	7	498	IHC	Yes (OS, stage, recurrence, mts)
Glioma	[119]	21	1535	IHC, WB, RT-PCR, DNA microarray	Yes (OS, PFS)
	[120]	11	740	IHC, WB, RT-PCR	Yes (OS, PFS)
Glioblastoma	[121]	10	715	IHC	Yes (OS, PFS)
Breast cancer	[122]	13	1734	Not reported	Yes (OS, DFS, DG, ER, PR, HER2, LN mts) No (age, tumor size)
Colorectal cancer	[123]	28	4546	IHC, PCR	Yes (OS, DFS)
	[124]	15	2297	IHC	Yes (OS, DFS) No (histology type, LN mts, distant mts)
	[125]	37	5397	IHC	Yes (OS, DFS, distant mts, invasion)
	[126]	12	3652	IHC	Yes (OS) No (LN mts, DG)
Esophageal cancer	[127]	5	451	IHC	No (OS)
Gastric cancer	[128]	8	603	IHC	Yes (OS, stage, mts)
	[129]	10	1569	IHC	Yes (OS, stage, invasion, mts)
Head and neck squamous cell carcinoma	[130]	22	2143	IHC	Yes (OS, DFS)
Renal cell carcinoma	[131]	4	611	IHC	Yes (CSS) No (OS, DFS, PFS)
Hepatocellular carcinoma	[132]	21	2592	IHC	Yes (OS, DFS, DG, stage) No (hepatitis, cirrhosis, α-fetoprotein level, tumor size, mts)
	[133]	10	890	IHC, RT-PCR, WB	Yes (OS, DFS, DG, α-fetoprotein level) No (tumor size, hepatitis, cirrhosis)
	[134]	15	1807	IHC	Yes (DG)
Non-small cell lung cancer	[135]	32	3595	IHC, RT-PCR	Yes (OS (only Asian patients), differentiation, LN mts) No (age, smoking status, stage, distant mts)
	[136]	13	1727	IHC, RT-PCR	Yes (OS, DG) No (DFS, gender, smoking status, invasion, mts, DG)
	[137]	11	1004	IHC	Yes (OS, stage, DG)
	[138]	23	2538	IHC, PCR	Yes (OS, LN mts) No (DFS)
Potentially malignant disorders of oral mucosa	[139]	2	251	IHC	Yes (risk of malignant transformation)
Ovarian cancer	[140]	8	1129	IHC	Yes (FIGO stage, DG) No (OS, DFS)
	[141]	17	1600	IHC	Yes (FIGO stage, size, LN mts)
Pancreatic ductal adenocarcinoma	[142]	15	908	IHC	Yes (OS, stage, DG, LN mts)

Abbreviations: IHC—immunohistochemistry (including tissue microarrays and immunofluorescent staining); RT-PCR—real time polymerase chain reaction; OS—overall survival; PFS—progression-free survival; DFS—disease-free survival; CSS—cancer-specific survival; ER—estrogen receptor; PR—progesterone receptor; mts—metastasis; DG—differentiation grade; LN—lymph node. * For OS, DFS, PFS, negative correlations are indicated in the table column.
